# Impact of childhood maltreatment on adult resilience

**DOI:** 10.1186/s12888-023-05124-w

**Published:** 2023-08-30

**Authors:** Chao Li, Guanyi Lv, Bangshan Liu, Yumeng Ju, Mi Wang, Qiangli Dong, Jinrong Sun, Xiaowen Lu, Liang Zhang, Ping Wan, Hua Guo, Futao Zhao, Mei Liao, Yan Zhang, Lingjiang Li, Jin Liu

**Affiliations:** 1 Department of Psychiatry, National Center for Mental Disorders, National Clinical Research Center for Mental Disorders, The Second Xiangya Hospital of Central South University, Changsha, 410011 Hunan China; 2 Department of Mental Health Center, Xiangya Hospital, Central South University, Changsha, China; 3 Department of Psychiatry, Lanzhou University Second Hospital, Lanzhou, China; 4 Affiliated WuTaiShan Hospital of Medical College of Yangzhou University, Yangzhou mental health centre, Yangzhou, 225003 Jiangsu China; 5 Affiliated Wuhan Mental Health Center, Huazhong University of Science and Technology, Wuhan, China; 6 Zhumadian Psychiatric Hospital, Zhumadian, 463000 Henan China

**Keywords:** Childhood maltreatment, Resilience, MDD, Depression

## Abstract

**Background:**

Previous studies suggested that childhood maltreatment is associated with poor health outcomes. While not everyone who experiences abuse as a child goes on to experience poor mental health, some traumatized people are grown to be more resilient than others. Few studies have examined the association between childhood maltreatment and adult resilience. This study aimed to determine different relationships between specific types and features of childhood maltreatment with adult resilience among Chinese with Major Depressive Disorder (MDD) and healthy controls (HCs).

**Methods:**

A total of 101 patients with MDD and 116 participants in the healthy control (HC) group from Zhumadian Psychiatric Hospital and its nearby communities were included in this analysis. Childhood maltreatment was assessed retrospectively using Childhood Trauma Questionnaire (CTQ). Adults’ resilience was assessed by the Connor-Davidson Resilience Scale (CD-RISC). Generalized linear models were applied between childhood maltreatment (specific types and features) and resilience adjusting for covariates.

**Results:**

The total score of CD-RISC and factor scores of strength, optimism, and tenacity in the HC group were higher than those in the MDD group. CTQ total score had a negative association with optimism score among participants in MDD (β=-0.087, P < 0.001) and HC (β=-0.074, P = 0.023) groups. Higher emotional neglect (EN) score (β=-0.169, P = 0.001) and physical neglect (PN) score (β=-0.153, P = 0.043) were related to a worse optimism score in MDD group. Emotional abuse (EA) score was associated with a worse tenacity score (β=-0.674, P = 0.031) in MDD group. For participants in HC group, higher EN and PN scores were related to worse resilience scores (tenacity, strength, and optimism).

**Conclusions:**

Patients with MDD showed lower optimism than HCs. Childhood maltreatment, especially childhood negect, independently contributed to optimism, with more severe childhood maltreatment predictive of worse performance of optimism. EA in childhood was also linked to worse tenacity in adult patients with MDD.

## Background

Childhood maltreatment is regarded as the abuse and neglect of children under 16 years, including physical and emotional abuse and neglect, and sexual abuse, which are related to long-term physical and mental health outcomes across the life course. A study suggested that females with mental illness had a higher rate of childhood sexual abuse than those in the general group [1]. Representative studies from large national American samples [2, 3] showed that American children were exposed to various types of violence in their childhood, including maltreatment, bullying, property victimization, sexual victimization, and eye-witness experience. Childhood maltreatment is commonly reported in individuals from developing countries, such as China. A systematic review reported that 26.6% of children under 18 years of age had suffered from physical abuse, 8.7% from sexual abuse, 19.6% from emotional abuse, and 16% from neglect in China [4]. It is reported that 36.6% of Chinese experienced physical abuse in their childhood [5]. A total of 24.8% and 17.6% of male and female college students experience sexual abuse in their childhood [6]. Previous studies showed that childhood maltreatment had an association with later psychiatric symptomatology, including major depressive disorder [7], posttraumatic stress disorder [8], and bipolar disorder [1]. In this case, childhood maltreatment is a major public problem.

Many children are at high risk of exposure to violence and its negative outcomes, so it is important to recognize how many children could successfully navigate these adverse events [9]. However, not everyone who experiences abuse as a child goes on to experience poor mental health [10]. Some people are or grow to be more resilient than others [11]. This observation had led many researchers to test the specific protective factors, including individual capacity for resilience, or the ability to face adversities or challenges successfully [12]. Resilience is defined as the ability to bounce back from setbacks [13], learn from mistakes [14], find inspiration in obstacles, and have faith that you can get through any stress or struggle you face in life [15, 16]. Previous studies focused on resilience capacity at individual-level, which is defined as one’s degree of their personal qualities, including adaptability, self-confidence, and ability to endure stress [17, 18]. Resilience capacity might be one of the factors that contribute to the recovery process after experiencing adversity and could decrease the risk of developing negative consequences after adverse events [19, 20]. It is important to explore how childhood maltreatment may have effects on resilience and compare the differences between individuals with and without psychotic disorders, such as Major Depressive Disorder (MDD), and this could provide recommendations for future public health interventions and for promoting public mental health.

Previous research suggested that childhood maltreatment could have detrimental effects on self-reported resilience in adulthood. A study conducted in the USA showed that childhood maltreatment had a relationship with worse resilience capacity among individuals in the community [21]. Another study from America revealed that exposure to violence in childhood was related to lower resilience capacity, however, this association was no longer statistically significant after adjusting for the symptoms of depression and anxiety [9]. A recent study using Atlanta’s large community-based sample reported that childhood emotional abuse and co-occurrence of childhood maltreatment might be deleterious to resilience in adulthood [22]. Most previous studies were conducted on data from high-income countries, including the USA. Few studies explored the association between childhood maltreatment and adult resilience in developing countries like China. There is value in conducting an analysis of relationships of childhood maltreatment to adult resilience in a new cultural and social setting. Besides, comparing the differences of associations between childhood maltreatment and adult resilience among patients with and MDD and healthy controls (HCs) might help to find the specific interventions among patients to promote their mental health.

In this study, we aim to investigate the association between childhood maltreatment and adult resilience capacity and compare differences among patients with MDD and HCs. Specifically, we intended to test whether the following childhood maltreatment features were related to worse resilience capacity (certain types of resilience capacity, including tenacity, strength, and optimism) in adulthood: (a) overall childhood maltreatment; (b) specific types of childhood maltreatment; (c) number of specific types of childhood maltreatment.

## Methods

### Sample and procedure

The data in this analysis was derived from the baseline data from a longitudinal project conducted in Zhumadian Psychiatric Hospital (Henan, China) and its nearby communities, and the project aimed to scrutinize the biological and psychological mechanisms of MDD. The enrolment procedure was set up in January 2013 and ended in December 2018. Two well-trained psychiatrists supervised the whole process and an eligibility criterion was set for the procedure for the two groups separately. The investigation was carried out following the latest version of the Declaration of Helsinki. The study design was reviewed and approved by the Medical Ethics Committees of the Second Xiangya Hospital of Central South University (S238) and the Zhumadian Psychiatric Hospital (S002). This clinical research has been registered at the Chinese Clinical Trial Registry, and the Registration number is ChiCTR1800014591.

The inclusion criteria for the two groups in the longitudinal study at baseline were as follows. For participants included in the MDD group, they (1) aged 18–60 years old, (2) diagnosed with MDD which was confirmed by two well-trained psychiatrists using the Structured Clinical Interview for the fourth edition of the Diagnostic and Statistical Manual of Mental Disorders (DSM-IV), (3) were medication free for ≥ 14 days, (4) currently had at least moderate depression severity, with a score $\ge$20 in the 24-item Hamilton Depression Rating Scale (HAMD_24_), (5) with no other psychiatric disorders in the past or present (except generalized anxiety disorder). For participants included in the HC group, they (1) aged 18–60 years old, (2) scored < 8 in the HAMD_24_, (3) had no current or lifetime substance abuse or diagnosis of any psychiatric disorders. For any participants, if they (1) had a history of systemic medical condition, neurological disorders or head injury, (2) are pregnant or breastfeeding, (3) had any other DSM-IV psychiatric disorder or alcohol/drug dependence, (4) had completed any similar neurocognitive assessments last year, (5) had color-blindness, or (6) had suicide ideation or suicidal behaviors, they were excluded from the Zhumadian longitudinal project. Any participants who had not completed childhood maltreatment or resilience measurements were not included in the current study. After excluding ineligible participants, a total of 101 patients with MDD and 116 participants in the HC group were included in this analysis. Informed consent of the participants was obtained after the nature of the procedures had been fully explained.

### Measures

#### Resilience

Resilience was assessed using the Chinese version of Connor-Davidson resilience scale (CD-RISC), which showed good validity and reliability among Chinese [23]. The Cronbach’s α for CD-RISC in this study was 0.902, indicating good internal consistency. The self-report questionnaire includes 25 items that could be grouped into three factors: tenacity, strength, and optimism [24]. Participants’ responses to each item ranged from 0 (completely disagree) to 4 (completely agree).

#### Childhood maltreatment

Childhood maltreatment was assessed by the Childhood Trauma Questionnaire (CTQ). CTQ consists of five factors (emotional abuse, EA; physical abuse; PA, sexual abuse, SA; emotional neglect, EN; physical neglect, PN) of maltreatment with 28 items in the questionnaire. The questionnaire is a retrospective assessment tool for assessing maltreatment before the age of 16 years old. The Chinese version of CTQ had shown good validity and reliability among the Chinese population [25]. The Cronbach’s α for CTQ in this study was 0.704, indicating good internal consistency. According to previous studies [26], we set the cutting scores of CTQ’s five factors to distinguish the participants with positive CTQ factors (exceeding the cutting scores) as follows: PA > 9, PN > 9, EA > 12, EN > 14, and SA > 7. The summary and factor scores of CTQ and the counts of CTQ factors exceeding the cutting scores were used for analysis.

#### Covariates

Potential confounders requiring adjustment were demographic factors (age, sex, and educational years), clinical information (total history of MDD and episode counts), and participants’ depression and anxiety rating scores.

The participants’ depression was assessed using HAMD_24_ which is a generally used clinician-rated scale [27]. HAMD_24_ had shown good reliability and validity in the Chinese population. A total of 12 items were rated from 0 to 4, three items were rated from 0 to 3, and nine items were scored from 0 to 2. The total score of the questionnaire ranges from 0 to 75 with a cutoff score of 20. Participants with a score of HAMD_24_$\ge$20 were regarded as having moderate depression [28].

We used a clinician-rated 14-item Hamilton Anxiety Rating Scale (HAMA_14_) to assess the participants’ anxiety symptoms [29]. HAMA_14_ presented good reliability and validity among the Chinese community population [30]. Each item of the questionnaire was rated from 0 to 4, and the total score ranges from 0 to 56.

### Statistical analysis

Mean (standard deviation) was used to summarize participants’ age and other continuous factors. Number (percentage) was reported to summarize participants’ sex. χ^2^ tests were used to test the group difference for categorical variables. *t*-test and *Mann-Whitney* test were used for examining the difference for continuous variables. With the adjustment of age, sex, and education years, we applied a 2 × 2 analysis of covariance (ANCOVA) of the diagnosis of MDD and childhood maltreatment on summary and factor scores of CD-RISC. Generalized linear models (GLMs) were applied to analyze the relationships between childhood maltreatment and resilience by adjusted models 1–2. The adjusted model 1 with adjustment of age, sex, and education, and adjusted model 2 was adjusted for age, sex, education, HAMD_24_, HAMA_14_, total history, and episode counts. The GLMs were applied separately for participants in MDD and HC groups. Statistical analyses were tested by SPSS 22.0. Two-tail *P* values < 0.05 were considered statistically significant in the analyses.

## Results

### Descriptive statistics

The demographics and clinic information of included participants were presented in Table 1. The educational years of the MDD group (11 years) are shorter than that of the HC group (12 years). For participants in the MDD group, 7 (6.9%) experienced EA in their childhood, 10 (9.9%) experienced SA in their childhood, 32 (31.7%) were exposed to EN in their childhood, 48 (47.5%) experienced PN in their childhood, and 7 (6.9%) experienced PA in their childhood, according to the results of CTQ. According to the CTQ scores, 46 (45.5%) participants in MDD did not experience any childhood maltreatment, 21 (20.8%) experienced one type of childhood maltreatment, 26 (25.7%) experienced two types of childhood maltreatment, 5 (5.0%) were exposed to three types of childhood maltreatment, and 3 (3.0%) experience four types of childhood maltreatment. For participants in the HC group, 6 (5.2%) experienced EA in their childhood, 7 (6.0%) were exposed to PA in their childhood, 43 (37.1%) experienced PN in their childhood, 27 (23.3%) experienced EN in their childhood, and 10 (8.6%) experienced SA in their childhood. According to the results of CTQ, 61 (52.6%) participants in the HC group did not experience any childhood maltreatment, 29 (25.0%) experienced one type of childhood maltreatment, 18 (15.5%) experienced two types of childhood maltreatment, 5 (4.3%) experienced three types of childhood maltreatment, 2 (1.7%) experienced four types of childhood maltreatment, and 1 (0.9%) experienced five types of childhood maltreatment. Table 2 showed the differences in CTQ scores and CD-RISC scores between MDD and HC groups. Factor scores of EA and PN in the MDD group were higher than those in the HC group. The total score of CD-RISC and factor scores of strength, optimism, and tenacity in the HC group were higher than those in the MDD group.

**Table 1:** Demographics and clinical information of major depressive disorder (MDD) and healthy control (HC) groups

Variables	MDD		*P*_*1*_	HC		*P*_*2*_	*P*_*3*_
	No CM (*n* = 44)	With CM (*n* = 57)		No CM (*n* = 61)	With CM (*n* = 55)		
Age (years)	34.43 (9.11)	33.68 (8.64)	0.677	30.52 (7.70)	33.69 (7.82)	**0.030**	0.081
Gender (Male)	22 (50%)	24 (42.11%)	0.430	39 (63.93%)	29 (52.73%)	0.221	0.054
Education (years)	11.30 (3.55)	9.98 (3.32)	0.061	12.69 (3.31)	10.87 (3.17)	**0.003**	**0.007**
HAMD_24_	33.00 (7.08)	34.84 (6.99)	0.196	1.05 (1.74)	1.67 (1.76)	0.058	**< 0.001**
HAMA_14_	18.59 (6.78)	19.07 (6.95)	0.728	0.82 (1.66)	1.27 (1.47)	0.122	**< 0.001**
Total history	46 (57)	38 (47)	0.455	-	-	-	-
Episode counts	2 (2)	2 (1)	0.315	-	-	-	-

Note: χ^2^ test (categorical variables)or *t* test (continuous variables) to explore the differences between different groups; data are presented as mean (standard deviation) or number (percentage); bold value indicates statistical significance; CM, childhood maltreatment; HAMA_14_, 14-item Hamilton Anxiety Rating Scale; HAMD_24_, 24-item Hamilton Rating Scale for Depression; *P*_1_, statistical significance for patients with MDD in different CM groups; *P*_2_, statistical significance for HCs in different CM groups; *P*_3_, statistical significance for patients in MDD and HCs.

**Table 2:** Scores of CTQ and CD-RISC between major depressive disorder (MDD) and healthy control (HC) groups

Variables	MDD (*n* = 101)	HC (*n* = 116)	*P*
Scores of CTQ			
Total scores	40.92 (10.67)	38.41 (10.74)	0.086
Emotional abuse	7.80 (2.63)	7.03 (2.68)	**0.033**
Physical abuse	6.00 (1.79)	6.08 (2.14)	0.774
Sexual abuse	5.44 (1.26)	5.59 (1.54)	0.435
Emotional neglect	12.08 (5.58)	11.08 (4.86)	0.159
Physical neglect	9.60 (3.51)	8.64 (3.24)	**0.036**
Trauma count	1 (1)	1 (1)	0.198
Scores of CD-RISC			
Total scores	66.61 (15.95)	86.48 (14.76)	**< 0.001**
Tenacity	34.06 (8.71)	43.13 (7.84)	**< 0.001**
Strength	21.98 (6.08)	29.99 (6.15)	**< 0.001**
Optimism	10.57 (2.68)	13.36 (3.72)	**< 0.001**

Note: Data are presented as mean (standard deviation); bold value indicates statistical significance

### Effects of diagnosis of MDD and childhood maltreatment on CD-RISC total and factor scores

Table 3 showed the results of a 2 × 2 ANCOVA (factor 1: diagnosis of MDD and factor 2: childhood maltreatment) on CD-RISC total and factor scores with the adjustment of age, gender, and educational years. There is no significant two-way interaction effect of a diagnosis of MDD and childhood maltreatment found for CD-RISC total and factor scores. Different main effects of a diagnosis of MDD were tested on CD-RISC total and factor scores. As for the main effect of childhood maltreatment, it presented a statistically significant difference in optimism scores, the difference was 7.179 (*P* = 0.006).

**Table 3:** Analysis of covariance (ANCOVA) of subscale scores of CD-RISC with age, gender, and education controlled

CDRISC	Major Depression Disorder			Health Control		*F*_*1*_	*P*_*1*_	Main effects of diagnosis		Main effects of CM		Interaction effects (CM and diagnosis)	
	No CM (n = 44)	With CM (n = 57)		No CM (n = 61)	With CM (n = 55)			*F*_*2*_	*P*_*2*_	*F*_*2*_	*P*_*2*_	*F*_*2*_	*P*_*2*_
Total scores	67.77 (15.37)	65.72 (16.47)		90.46 (13.05)	82.07 (15.39)	28.816	**< 0.001**	79.783	**< 0.001**	3.699	0.056	2.319	0.129
Tenacity	34.61 (8.34)	33.63 (9.03)		45.26 (7.15)	40.76 (7.95)	20.628	**< 0.001**	55.563	**< 0.001**	3.354	0.068	2.525	0.114
Strength	21.95 (5.96)	22.00 (6.22)		31.03 (4.60)	28.84 (7.38)	27.920	**< 0.001**	81.651	**< 0.001**	0.653	0.420	1.599	0.207
Optimism	11.20 (2.72)	10.09 (2.57)		14.16 (4.40)	12.47 (2.54)	14.039	**< 0.001**	32.646	**< 0.001**	7.719	**0.006**	0.495	0.482

Note: Data are presented as mean (standard deviation); bold value indicates statistical significance; CM, childhood maltreatment; *F*_1_, F test value for corrected model; *P*_1_, statistical significance of corrected model; *F*_2_, F test value for main effects of MDD; *P*_2_, statistical significance for main effects of MDD; *F*_3_, F test value for main effect of CM; *P*_3_, statistical significance of main effect of CM; *F*_4_, F test value for interaction effect between CM and MDD; *P*_4_, statistical significance of interaction effect between CM and MDD.

### Association between CTQ total score and CD-RISC factor scores

The results of adjusted GLMs were presented in Tables 4 and 5. In adjusted model 1, CTQ total score had a negative association with optimism score (*β=*-0.081[95%CI,-0.129 to -0.033], *P* = 0.001) among participants in the MDD group, and similar results were found in adjusted model 2. For participants in the HC group, CTQ total score had a negative association with tenacity score (*β=*-0.005[95%CI,-0.008 to -0.002], *P* = 0.001) and optimism score (*β=*-0.007[95%CI,-0.012 to -0.002], *P* = 0.010) in adjusted model 1. After adjusting for age, sex, educational years, HAMD_24_, and HAMA_14_, similar associations were found in adjusted model 2 in Table 5.

**Table 4:** Generalized linear models of childhood maltreatment on CD-RISC subscale scores in the MDD group (*n* = 101)

	Tenacity		Strength		Optimism	
	*β* (95% CI)	*P*	*β* (95% CI)	*P*	*β* (95% CI)	*P*
Model 1 (Adjusted for age, sex, and education)						
Total scores	-0.098 (-0.253, 0.057)	0.216	-0.087 (-0.199, 0.024)	0.125	-0.081 (-0.129, -0.033)	**0.001**
Emotional abuse	-0.643 (-1.262, -0.024)	**0.042**	-0.433 (-0.882, 0.016)	0.059	-0.181 (-0.381, 0.020)	0.078
Physical abuse	0.125 (-0.776, 1.026)	0.785	-0.099 (-0.750, 0.552)	0.089	-0.148 (-0.437, 0.141)	0.317
Sexual abuse	0.248 (-1.021, 1.518)	0.702	0.472 (-0.441, 1.385)	0.311	-0.130 (-0.538, 0.279)	0.533
Emotional neglect	-0.127 (-0.423, 0.169)	0.705	-0.185 (-0.397, 0.027)	0.086	-0.169 (-0.259, -0.079)	**0.001**
Physical neglect	-0.279 (-0.744, 0.186)	0.239	-0.126 (-0.463, 0.211)	0.465	-0.153 (-0.301, -0.005)	**0.043**
Trauma count	-0.322 (-1.853, 1.188)	0.699	-0.423 (-1.520, 0.673)	0.449	-0.489 (-0.970, -0.008)	**0.046**
Model 2 (Adjusted for age, sex, education, HAMD_24_, HAMA_14_, total history, and episodes)						
Total scores	-0.096 (-0.249, 0.057)	0.217	-0.071 (-0.183, 0.041)	0.213	-0.087 (-0.133, -0.040)	**< 0.001**
Emotional abuse	-0.674 (-1.286, -0.062)	**0.031**	-0.383 (-0.834, 0.068)	0.096	-0.191 (-0.390, 0.008)	0.060
Physical abuse	0.117 (-0.803, 1.038)	0.803	0.030 (-0.642, 0.702)	0.930	-0.165 (-0.461, 0.132)	0.276
Sexual abuse	0.347 (-0.921, 1.615)	0.591	0.700 (-0.216, 1.616)	0.134	-0.131 (-0.541, 0.279)	0.531
Emotional neglect	-0.121 (-0.410, 0.168)	0.412	-0.174 (-0.383, 0.035)	0.103	-0.176 (-0.264, -0.089)	**< 0.001**
Physical neglect	-0.263 (-0.719, 0.193)	0.259	-0.086 (-0.421, 0.248)	0.613	-0.164 (-0.309, -0.019)	**0.027**
Trauma count	-0.403 (-1.921, 1.114)	0.602	-0.271 (-1.379, 0.836)	0.631	-0.579 (-1.057, -0.100)	**0.018**

Note: Bold value indicates statistical significance; CM, childhood maltreatment

**Table 5:** Generalized linear models of childhood maltreatment on CD-RISC subscale scores in HC group (*n* = 116)

	Tenacity		Strength		Optimism	
	*β* (95% CI)	*P*	*β* (95% CI)	*P*	*β* (95% CI)	*P*
Model 1 (Adjusted for age, sex, and education)						
Total scores	-0.005 (-0.008, -0.002)	**0.001**	-0.002 (-0.006, 0.001)	0.120	-0.007 (-0.012, -0.002)	**0.010**
Emotional abuse	-0.004 (-0.015, 0.006)	0.410	-0.001 (-0.014, 0.011)	0.817	-0.012 (-0.031, 0.008)	0.237
Physical abuse	0.000 (-0.014, 0.013)	0.963	0.009 (-0.006, 0.024)	0.254	-0.005 (-0.030, 0.019)	0.662
Sexual abuse	0.005 (-0.012, 0.023)	0.553	0.019 (-0.001, 0.040)	0.065	-0.002 (-0.034, 0.031)	0.910
Emotional neglect	-0.016 (-0.022, -0.009)	**< 0.001**	-0.011 (-0.019, -0.004)	**0.004**	-0.017 (-0.029, -0.006)	**0.003**
Physical neglect	-0.019 (-0.028, -0.010)	**< 0.001**	-0.014 (-0.025, -0.003)	**0.012**	-0.025 (-0.042, -0.008)	**0.003**
Trauma count	-0.037 (-0.065, -0.010)	**0.008**	-0.008 (-0.041, 0.024)	0.614	-0.053 (-0.104, -0.002)	**0.040**
Model 2 (Adjusted for age, sex, education, HAMD_24_, HAMA_14_, total history, and episodes)						
Total scores	-0.171 (-0.302, -0.041)	**0.010**	-0.058 (-0.164, 0.048)	0.282	-0.074 (-0.138, -0.010)	**0.023**
Emotional abuse	-0.059 (-0.570, 0.451)	0.819	0.025 (-0.380, 0.431)	0.903	-0.106 (-0.353, 0.141)	0.399
Physical abuse	0.012 (-0.628, 0.652)	0.971	0.307 (-0.199, 0.813)	0.235	-0.062 (-0.372, 0.249)	0.697
Sexual abuse	0.326 (-0.565, 1.217)	0.473	0.660 (-0.039, 1.359)	0.064	-0.013 (-0.446, 0.420)	0.953
Emotional neglect	-0.597 (-0.890, -0.304)	**< 0.001**	-0.292 (-0.535, -0.049)	**0.018**	-0.198 (-0.345, -0.050)	**0.009**
Physical neglect	-0.716 (-1.138, -0.293)	**0.001**	-0.378 (-0.723, -0.033)	**0.033**	-0.299 (-0.507, -0.091)	**0.005**
Trauma count	-1.286 (-2.599, 0.027)	0.055	-0.082 (-1.142, 0.978)	0.880	-0.586 (-1.225, 0.052)	0.072

Note: Bold value indicates statistical significance; CM, childhood maltreatment

### Association between CTQ factor scores and CD-RSIC factor scores

In Table 4, among participants in the MDD group, adjusted model 1 showed negative associations between factor scores of EA and tenacity score (*β=*-0.643[95%CI,-1.262 to -0.024], *P* = 0.042), higher EN score was related to worse optimism score (*β=*-0.169[95%CI,-0.259 to -0.079], *P* = 0.001), and higher PN score was associated with worse optimism score (*β=*-0.153[95%CI,-0.301 to -0.005], *P* = 0.043). The associations which were found in adjusted model 1 among patients with MDD were statistically significant in adjusted model 2. For participants in the HC group, a higher EN score was associated with a worse tenacity score (*β=*-0.016 [95%CI,-0.022 to -0.009], *P* < 0.001), strength score (*β=*-0.011 [95%CI,-0.019 to -0.004], *P* = 0.004), and optimism score (*β=*-0.017 [95%CI,-0.029 to -0.006], *P* = 0.003) with the adjustment of age, sex, and educational years. Negative associations were also found between factor scores of PN and tenacity score (*β=*-0.019 [95%CI,-0.028 to -0.010], *P* < 0.001), strength score (*β=*-0.014 [95%CI,-0.02 5to -0.003], *P* = 0.012), and optimism score (*β=*-0.025 [95%CI,-0.042 to -0.008], *P* = 0.003) in adjusted model 1. The associations which were found in adjusted model 1 were statistically significant in adjusted model 2.

### Association between counts of positive CTQ factors and CD-RISC factor scores

For patients with MDD, a negative association between counts of positive CTQ factors and optimism scores was significant in both adjusted model 1 (*β=*-0.489[95%CI,-0.970 to -0.009], *P* = 0.046) and adjusted model 2 (*β=*-0.579[95%CI,-1.057 to -0.100], *P* = 0.018). However, negative associations between counts of positive CTQ factors and tenacity scores (*β=*-0.037[95%CI,-0.065 to -0.010], *P* = 0.008) and optimism scores (*β=*-0.053[95%CI,-0.104 to -0.002], *P* = 0.040) were only statistically significant in adjusted model 1.

## Discussion

Previous studies conducted in China focused on testing the mediating roles of resilience on the relationship between childhood maltreatment and depression [31–33], mainly among youth [34] and adolescents [35]. Our study focused on the relationship between childhood maltreat and resilience among patients with MDD and HCs in adulthood by using baseline data of a longitudinal study. This study found that the specific features of childhood maltreatment had different associations with resilience capacity in adult patients with MDD and HCs. Participants in the HC group had higher overall and factor scores of CTQ than participants in the MDD group. Child neglect had detrimental effects on tenacity, strength, and optimism among participants in the HC group, however, a negative association was found between optimism and child neglect for patients with MDD. EA was negatively associated with tenacity for patients with MDD. This study provides evidence for further understanding of the relationship between CM and adult resilience in patients with MDD and HCs.

We found that participants in the MDD group showed lower optimism scores than those in the HC group. Previous studies suggested that the interactive association between optimism and depression, individuals experiencing high levels of depression symptoms reduced their optimism bias when they met with a life event [36], meanwhile, people with low levels of dispositional optimism would have more harmful or dysfunctional expectations about their future [37]. This may suggest that optimism could have a protective function against adverse mental health outcomes, and the underlying mechanism could be that individuals with high optimism were more likely to exert efforts to manage stress actively and less likely to disengage in the face of adversities [38]. Childhood maltreatment was associated with worse optimism among individuals in both MDD and HC groups. This is along with previous studies [39, 40] showing that childhood maltreatment was linked to low dispositional optimism. One possible explanation might be that childhood maltreatment changes children’s cognitive beliefs and even their ability to have positive mental imagery of the future [39]. Besides, the effect size of the relationship between childhood maltreatment and optimism among participants in the MDD group is higher than that in the HC group. Childhood neglect, including PN and EN, was linked to adults’ worse optimism, tenacity, and strength for participants in the HC group. But for patients with MDD, a negative association was only found between child neglect and adults’ optimism. This is different from the conclusion of Chen’s study [41], in which they suggested that neglect is often unintentional and may not have deleterious effects on optimism as abuse. This is probably because Chen’s study was conducted on data from the PTSD population, which have differences from participants in our analysis. Besides, the possible explanation on the results that the negative associations between EN and PN, and tenacity disappeared in patients with MDD were the genetic effects on resilience [42], patients with MDD might be with lower tenacity capacity with the cause of some genetic reasons, instead of the effects of childhood maltreatment. Our results suggested that promotion optimism might be an effective way to lessen the likelihood of MDD development and severity in people with childhood maltreatment experience.

EA in childhood had an association with worse tenacity among patients with MDD. This finding is similar to two previous studies, one of which reported that EA had the highest magnitude of impact on resilience after adjusting for psychological distress; and another study [43] presented that associations between EA and higher levels of negative traits and lower levels of positive traits. The possible explanations were as follows: EA is known to disrupt individuals’ development of self-concept, impairing emotion regulation, and leading to negative self-perceptions [44], and this psychological impact might be harmful to long-term adjustment, reducing their confidence when facing challenges [22]. Since EA might be more chronic than other kinds of maltreatment, which could be more deleterious for future psychological function [22].

Along with the results of studies among the general population, our analysis showed that childhood maltreatment was related to tenacity only in the HC group. Poole et al. 2017 [45] suggested that a stronger relationship between childhood maltreatment and depression existed among individuals with low resilience than among those with high resilience. Resilience could be regarded as a dynamic personality trait that may be enhanced through practice and training. It is important to figure out the high risk of the population with low resilience, such as adults with childhood maltreatment, and provide resilience-training programs (e.g. The Penn Resiliency Program) for these target individuals. It was reported that resilience-training programs could foster personal characteristics [46] and had been shown to improve the rate of depressive symptoms [47].

The number of positive CTQ factors was only significantly associated with lower optimism among patients with MDD. Besides, counts of positive CTQ factors were no longer significantly related to optimism after controlling for current depression and anxiety severity in the general population. Results from previous cross-sectional studies showed that the negative associations between the number of childhood maltreatment types [48] or adverse childhood events [45] and resilience capacity were small among the general population. The general population included in our analysis was small, and it is possible that no significant association was tested in this small general sample. Unlike the previous study’s findings [22], our results did not suggest that the complexity of childhood maltreatment exposure with multiple types might be specifically noxious for adults’ resilience. Our results may indicate that cumulative adversity models are limited since the models assumed that adverse effects across childhood maltreatment types were additive and equal. The assumption may not be appropriate [49]. More research with a larger sample should be conducted to examine the cumulative adversity models.

Our study has some clinical and practical implications. First, this study revealed that childhood maltreatment was a risk for poor performance of resilience in adult patients with MDD, suggesting the importance of identifying childhood maltreatment and assessments of resilience in the clinical management of MDD. Second, resilience training may be a promising and viable intervention for patients with MDD who exposures to childhood maltreatment when established treatment strategies. Additionally, general adults with childhood maltreatment should also be advised to take some resilience training in the community, in order to cope with the adverse impacts associated with childhood adversity. According to previous studies [50, 51], CBT can effectively increase resilience. When administering medication to patients with MDD, particularly those who have a history of childhood maltreatment, CBT may also be an alternative option to enhance resilience.

Strengths and limitations.

Previous research on the relationship between childhood trauma and resilience has focused on adolescents, with very little research on adults with MDD. The present study provides further evidence on the relationship between childhood trauma and resilience by including medication-free patients with MDD and HCs. To be more specific, based on a Chinese sample, this study explored the associations between various features of childhood maltreatment and summary and factor scores of resilience capacity in adulthood. Besides, this study compared the different relationships between patients with MDD and HCs to better understand the impacts of childhood maltreatment on resilience. However, there were several limitations in the present study. Firstly, this analysis was based on baseline data from a longitudinal study, inference on the causal relationship between childhood maltreatment and resilience could not be made. Further exploration based on longitudinal data was needed to examine these relationships. Second, the participants’ childhood maltreatment and resilience were self-reported, and recall bias may influence our results and conclusion. Third, the conclusion derived from this analysis was based on data that was from a psychiatric hospital and nearby community, which was not a representative sample of the whole Chinese population, it is unclear yet whether the findings could be proved in other population.

## Conclusion

In conclusion, the present study investigated the influence of childhood maltreatment on resilience in patients with MDD and HCs. We found that childhood maltreatment were the risk factors for lower optimism, and EA might be responsible for the poor performance of tenacity in patients with MDD. These findings highlight the importance of early identification of childhood maltreatment and specific training on resilience in the treatment course of MDD.

## Data Availability

All data generated or analyzed during this study are included in this article.
